# Subjective expectations from radiotherapy and chemotherapy in patients with oncological illnesses

**DOI:** 10.1192/j.eurpsy.2022.1691

**Published:** 2022-09-01

**Authors:** E. Rasskazova, M. Kovyazina, M. Karakurchi

**Affiliations:** 1 Mental Health Research Center, Medical Psychology, Moscow, Russian Federation; 2 Moscow State University, Clinical Psychology, Moscow, Russian Federation; 3 Lomonosov Moscow State University, Faculty Of Psychology, Moscow, Russian Federation; 4 Research Center of Neurology, Department Of Neurorehabilitation And Physiotherapy, Moscow, Russian Federation; 5 Lomonosov Moscow State University, Faculty Of Psychology, Московская обл, г Реутов, Russian Federation

**Keywords:** oncology, radiotherapy, chemotherapy

## Abstract

**Introduction:**

Expectations and fears about chemotherapy and radiotherapy in patients with oncological illness may not only affect their subjective well-being (Shaverdian et al., 2018) but also treatment satisfaction and complaints of side effects (Guidolin et al., 2018, Dong et al., 2014, Colagiuri et al., 2013).

**Objectives:**

The aim was to compare beliefs about treatment in patients referred to radiation therapy and chemotherapy, and to reveal their relationship to health anxiety and subjective well-being.

**Methods:**

53 patients referred to radiation therapy and 63 patients referred to chemotherapy completed the Treatment Perception in Oncological Illnesses Scale (Kovyazina et al., 2021), Illness and Treatment Self-Regulation Questionnaire (Kovyazina et al., 2019), Satisfaction with Life Scale (Diener et al., 1985) and Scale of Positive and Negative Experiences (Diener et al., 2009).

**Results:**

Compared to radiation therapy, with chemotherapy, patients tend to be more doubtful about the effectiveness of treatment and more anxious about the need for it (p<.05). Moderated mediation analysis demonstrated that lack of understanding, doubts about the effectiveness and anxiety about radiation and chemotherapy are associated with subjective ill-being indirectly - through a higher level of health anxiety (β=-.79--.35, SE=.17-.26, 95% CI [-1.42 - -.75 – -.37 - -0,08]). Feelings of helplessness regarding treatment mediated the relationship between doubts and confidence about treatment effectiveness and well-being in both groups.

**Conclusions:**

Results demonstrated that some fears and expectations about chemo- and radiotherapy could provoke health anxiety and helplessness regarding treatment that is related to poorer well-being.

**Disclosure:**

No significant relationships.

